# Impact of albuminuria on the various causes of death in diabetic patients: a nationwide population-based study

**DOI:** 10.1038/s41598-022-23352-0

**Published:** 2023-01-06

**Authors:** Semin Cho, Hyuk Huh, Sehoon Park, Soojin Lee, Sehyun Jung, Minsang Kim, Kyu-na Lee, Jin Hyuk Paek, Woo Yeong Park, Kyubok Jin, Seungyeup Han, Kwon Wook Joo, Chun Soo Lim, Yon Su Kim, Kyungdo Han, Yaerim Kim, Dong Ki Kim

**Affiliations:** 1 Department of Internal Medicine, Chung-Ang University Gwang-Myeong Hospital, Gwangmyeong-si, Korea; 2 Department of Internal Medicine, Inje University Busan Paik Hospital, Busan, Korea; 3 Department of Internal Medicine, Seoul National University College of Medicine, Seoul, Korea; 4 Department of Internal Medicine, Uijeongbu Eulji University Medical Center, Uijeongbu-si, Korea; 5 Department of Internal Medicine, Gyeongsang National University Hospital, Jinju-si, Korea; 6 Department of Internal Medicine, Republic of Korea Air Force Education and Training Command, Jinju-si, Korea; 7 Department of Biomedicine & Health Science, The Catholic University, Seoul, Korea; 8 Department of Internal Medicine, Keimyung University School of Medicine, Dalgubeol-Daero, Dalseo-Gu, Daegu, 103542601 Korea; 9 Department of Internal Medicine, Seoul National University Hospital, Seoul, Korea; 10 Department of Internal Medicine, Seoul National University Boramae Medical Center, Seoul, Korea; 11 Department of Statistics and Actuarial Science, Soongsil University, 369 Sangdo-Ro, Dongjak-Gu, Seoul, 06978 Korea; 12 Kidney Research Institute, Seoul National University Hospital, Seoul, Korea

**Keywords:** Endocrine system and metabolic diseases, Nephrology

## Abstract

Diabetes mellitus (DM) is a well-known risk factor for mortality, and the risk is exacerbated by coexisting diabetic kidney disease (DKD). We aimed to explore the impact of DM on each cause of mortality according to kidney function and the presence of albuminuria. Data on subjects with DM were extracted from the Nationwide Health Insurance Database of South Korea between 2009 and 2012. Subjects were divided by eGFR and albuminuria into five groups. To evaluate the risk of diabetes, we used the Cox proportional hazards model. A total of 2,614,662 patients were enrolled in this study. Most causes of death showed a higher incidence in an advanced stage of DKD. In addition to all-cause mortality and cardiovascular death, the risk of death from neoplasms and diseases of the endocrine, respiratory, and digestive systems is increased by albuminuria. The synergistic effect of a reduced eGFR and the presence of albuminuria was prominent in death from circulatory diseases, and endocrine and metabolic diseases. The risk for mortality was different according to the stage of DKD. Even in patients with a favorable eGFR, the presence of albuminuria significantly increased the risk for mortality, especially that due to cardiovascular causes.

## Introduction

Diabetes mellitus (DM) is one of the leading causes of disability and mortality, accounting for approximately 10% of all deaths between the ages of 20 and 79 years^1,2^. In addition, following the increase in prevalence of diabetes^3,4^, its disease burden notably increased with diverse aspects of the disease spectrum. As a multisystemic disorder that influences vascular injury with the dysregulation of blood glucose, diabetes significantly contributes to increased mortality^5^. In particular, the rates of premature mortality from diabetes increased by 5% between 2000 and 2016^6^.

DM is a well-known underlying risk factor for cardiovascular disease, including coronary heart disease, cerebrovascular disease, and peripheral arterial disease^7–10^. Additionally, it can affect the prognosis of such comorbidities, including malignant disease, cerebrovascular disease, Alzheimer’s disease, respiratory disease, and kidney disease ^11–14^. Although the pathophysiology is distinguished according to the disease category, diabetes has a common role as an independent risk factor for disease progression. In contrast to the diverse effect of diabetes on other conditions, there is a lack of literature identifying the impact of diabetes on various causes of mortality.

For patients with diabetes, the prognosis varies widely according to the presence of microvascular complications such as diabetic kidney disease (DKD). The presence of albuminuria has usually been regarded as an early marker of kidney damage as well as decreased kidney function, and it is closely linked to poor prognosis in diabetic patients^15,16^. The significance of albuminuria as a prognostic marker for major cardiovascular disease and mortality has been emphasized^17,18^. Moreover, albuminuria also has a role in increasing the risk of other systemic diseases, such as pulmonary disease, inflammatory bowel disease, and cerebrovascular disease^19–21^. However, the impact of albuminuria on each cause of death has not yet been clearly evaluated.

In this study, we aimed to elucidate the risk of each cause of mortality according to the stage of DKD. In addition, we tried to identify the impact of the presence of albuminuria on mortality within each stage of DKD.

## Results

### Baseline characteristics

**Figure 1:**
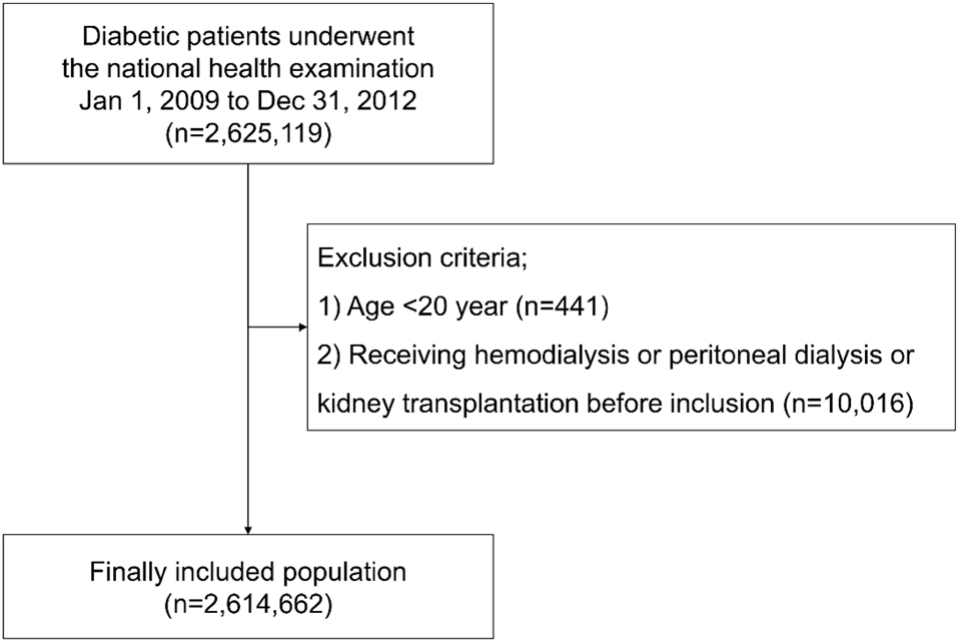
Flow diagram of the study.

Among 2,625,119 patients with diabetes who underwent the national health examination, 2,614,662 patients were ultimately enrolled in this study (Fig. 1). The comparison of baseline characteristics between the groups is shown in Table 1. The average age of the subjects in all groups was 50 years or older, and the proportion of males was higher than that of females, except in group 4. More than 50% of the study participants, except those in group 1, had hypertension. There was a significant difference in the overall demographic findings according to the DKD stage, but it was not stratified according to kidney function. The proportion of subjects with albuminuria increased with decreased kidney function.

**Table 1:** Baseline characteristics according to DKD stage.

	^a^No DKD (n = 880,927)	^b^DKD stage 1 (n = 51,406)	^c^DKD stage 2 (n = 1,384,982)	^d^DKD stage 3 (n = 257,638)	^e^DKD stage 4 (n = 39,709)	*P* value
Age (years)	53.18 ± 11.49	53.4 ± 11.06	58.31 ± 12.12	67.2 ± 9.98	57.63 ± 13.16	< 0.0001
20–29	22,776 (2.59)	999 (1.94)	11,910 (0.86)	301 (0.12)	679 (1.71)	
30–39	82,082 (9.32)	3889 (7.57)	74,648 (5.39)	1564 (0.61)	2927 (7.37)	
40–49	197,603 (22.43)	12,415 (24.15)	247,485 (17.87)	13,256 (5.15)	6926 (17.44)	
50–59	311,760 (35.39)	18,812 (36.59)	382,098 (27.59)	30,519 (11.85)	11,443 (28.82)	
60–69	194,836 (22.12)	11,227 (21.84)	382,166 (27.59)	94,576 (36.71)	9164 (23.08)	
≥ 70	71,870 (8.16)	4064 (7.91)	286,675 (20.70)	117,422 (45.58)	8570 (21.58)	
**Sex**						< 0.0001
Male	562,581 (63.86)	36,051 (70.13)	843,752 (60.92)	110,849 (43.03)	25,582 (64.42)	
Female	318,346 (36.14)	15,355 (29.87)	541,230 (39.08)	146,789 (56.97)	14,127 (35.58)	
BMI (kg/m^2^)	24.96 ± 3.51	25.54 ± 3.98	25.13 ± 3.81	25.12 ± 3.42	24.82 ± 3.37	< 0.0001
< 18.5	16,790 (1.91)	1,182 (2.30)	19,663 (1.42)	4417 (1.71)	704 (1.77)	
18.5 ≤ < 23	235,062 (26.68)	11,846 (23.04)	330,474 (23.86)	61,445 (23.85)	10,883 (27.41)	
23 ≤ < 25	215,368 (24.45)	11,082 (21.56)	346,881 (25.05)	63,275 (24.56)	10,175 (25.62)	
25 ≤ < 30	344,002 (39.05)	21,002 (40.86)	583,652 (42.14)	108,572 (42.14)	15,248 (38.40)	
≥ 30	69,705 (7.91)	6294 (12.24)	104,312 (7.53)	19,929 (7.74)	2699 (6.80)	
**Smoking history**						< 0.0001
Never smoker	447,417 (50.79)	22,913 (44.57)	770,372 (55.62)	182,650 (70.89)	21,030 (52.96)	
Ex-smoker	155,334 (17.63)	9674 (18.82)	268,535 (19.39)	40,583 (15.75)	9028 (22.74)	
Current smoker	278,176 (31.58)	18,819 (36.61)	346,075 (24.99)	34,405 (13.35)	9651 (24.30)	
**Alcohol consumption**						< 0.0001
Non-drinker	442,747 (50.26)	23,729 (46.16)	800,702 (57.81)	198,263 (76.95)	23,052 (58.05)	
Mild drinker	326,402 (37.05)	18,628 (36.24)	452,929 (32.70)	48,528 (18.84)	13,422 (33.80)	
Heavy drinker	111,778 (12.69)	9049 (17.60)	131,351 (9.48)	10,847 (4.21)	3235 (8.15)	
Regular physical activity	176,918 (20.08)	9580 (18.64)	290,988 (21.01)	48,711 (18.91)	8736 (22.00)	< 0.0001
**Income level**						< 0.0001
1st quartile	190,147 (21.58)	11,612 (22.59)	286,580 (20.69)	54,918 (21.32)	5927 (14.93)	
2nd quartile	188,211 (21.37)	11,428 (22.23)	254,189 (18.35)	42,492 (16.49)	5987 (15.08)	
3rd quartile	235,985 (26.79)	13,531 (26.32)	351,556 (25.38)	61,222 (23.76)	9530 (24.00)	
4th quartile	266,584 (30.26)	14,835 (28.86)	492,657 (35.57)	99,006 (38.43)	18,265 (46.00)	
Comorbidities						
Hypertension	429,176 (48.72)	32,721 (63.65)	793,557 (57.30)	200,840 (77.95)	24,793 (62.44)	< 0.0001
Dyslipidemia	332,360 (37.73)	24,353 (47.37)	586,012 (42.31)	136,630 (53.03)	17,665 (44.49)	< 0.0001
COPD	66,821 (7.59)	4070 (7.92)	130,013 (9.39)	35,915 (13.94)	3995 (10.06)	< 0.0001
Cancer	23,876 (2.71)	1411 (2.74)	44,187 (3.19)	11,247 (4.37)	1372 (3.46)	< 0.0001
CHF	1434 (0.16)	156 (0.30)	3525 (0.25)	2268 (0.88)	457 (1.15)	< 0.0001
Systolic BP (mmHg)	128.21 ± 15.50	133.15 ± 17.47	129.16 ± 15.75	130.68 ± 16.88	130.01 ± 16.8	< 0.0001
Diastolic BP (mmHg)	79.17 ± 10.21	82.10 ± 11.34	79.14 ± 10.25	78.20 ± 10.59	78.28 ± 10.48	< 0.0001
Total cholesterol (mg/dL)	196.54 ± 45.81	206.33 ± 54.47	198.06 ± 46.77	195.25 ± 49.49	191.99 ± 47.61	< 0.0001
HDL-C (mg/dL)	52.55 ± 33.41	51.78 ± 21.34	51.75 ± 22.52	50.41 ± 24.59	69.17 ± 108.30	< 0.0001
LDL-C (mg/dL)	111.46 ± 80.82	113.81 ± 75.29	113.62 ± 87.86	111.69 ± 85.67	110.23 ± 69.02	< 0.0001
Dipstick albuminuria ≥ 1 +	0 (0)	51,406 (5.51)	85,435 (6.17)	34,375 (13.34)	7659 (19.29)	< 0.0001

Data are presented as the mean ± standard deviation, or n(%).

*DKD* diabetic kidney disease; *BMI* body mass index; *COPD* chronic obstructive pulmonary disease; *CHF* congestive heart failure; *BP* blood pressure; *HDL-C* high-density lipoprotein cholesterol; *LDL-C* low-density lipoprotein cholesterol.

^a^No DKD: estimated glomerular filtration rate(eGFR) ≥ 90 mL/min/1.73m^2^, negative or trace in dipstick proteinuria, ^b^DKD stage 1: eGFR ≥ 90 mL/min/1.73m^2^, ≥ 1 + in dipstick proteinuria, ^c^DKD stage 2: 60 mL/min/1.73m^2^ ≤ eGFR < 90 mL/min/1.73m^2^, ^d^DKD stage 3: 30 mL/min/1.73m^2^ ≤ eGFR < 60 mL/min/1.73m^2^, ^e^DKD stage 4: eGFR < 30 mL/min/1.73m^2^.

### Risk of various causes of death according to DKD stage

The major causes of death were neoplasm and circulatory system diseases irrespective of kidney function. The more advanced the stage of DKD was, the higher the incidence rate for mortality with endocrine and metabolic diseases and genitourinary system disorders. For all categories of death except endocrine and metabolic disorders, the highest incidence rate occurred in DKD stage 3. Subjects with DKD stage 1 showed a higher incidence rate of most causes of death except nervous system disorders than subjects with no DKD (Table 2).

**Table 2:** Incidence rates of causes of death according to DKD stage.

	^a^No DKD (n = 880,927)		^b^DKD stage 1 (n = 51,406)		^c^DKD stage 2 (n = 1,384,982)		^d^DKD stage 3 (n = 257,638)		^e^DKD stage 4 (n = 39,709)		*P*-value
	N	IR (95% CI)	N	IR (95% CI)	N	IR (95% CI)	N	IR (95% CI)	N	IR (95% CI)	
All Cause	69,476	9.00 (8.93–9.07)	6501	14.66 (14.31–15.02)	159,776	13.15 (13.09–13.22)	66,850	31.45 (31.22–31.69)	8749	25.48 (24.95–26.02)	< 0.001
Infectious disease	1835	0.24 (0.23–0.25)	176	0.40 (0.34–0.46)	4313	0.36 (0.35–0.37)	2091	0.98 (0.94–1.03)	198	0.58 (0.50–0.66)	< 0.001
Neoplasm	25,891	3.35 (3.31–3.40)	2024	4.57 (4.37–4.77)	54,963	4.53 (4.49–4.56)	15,704	7.39 (7.27–7.51)	1587	4.62 (4.40–4.85)	< 0.001
Endocrine and metabolic disease	4487	0.58 (0.56–0.60)	684	1.54 (1.43–1.66)	12,101	1.00 (0.98–1.01)	8507	4.00 (3.92–4.09)	1855	5.40 (5.16–5.65)	< 0.001
Nervous system disorders	1510	0.20 (0.19–0.21)	87	0.20 (0.16–0.24)	4370	0.36 (0.35–0.37)	1877	0.88 (0.84–0.92)	129	0.38 (0.32–0.45)	< 0.001
Diseases of the circulatory system	11,121	1.44 (1.41–1.47)	1204	2.72 (2.57–2.87)	31,597	2.60 (2.57–2.63)	16,452	7.74 (7.62–7.86)	1909	5.56 (5.32–5.81)	< 0.001
Diseases of the respiratory system	5309	0.69 (0.67–0.71)	473	1.07 (0.98–1.17)	15,046	1.24 (1.22–1.26)	6529	3.07 (3.00–3.15)	533	1.55 (1.43–1.69)	< 0.001
Diseases of the digestive system	4664	0.60 (0.59–0.62)	474	1.07 (0.98–1.17)	6361	0.52 (0.51–0.54)	2308	1.09 (1.04–1.13)	305	0.89 (0.79–0.99)	< 0.001
Diseases of the genitourinary system	757	0.10 (0.09–0.11)	189	0.43 (0.37–0.49)	2892	0.24 (0.23–0.25)	3257	1.53 (1.48–1.59)	1166	3.40 (3.21–3.60)	< 0.001
Injury from external causes	8226	1.07 (1.04–1.09)	650	1.47 (1.36–1.58)	14,310	1.18 (1.16–1.20)	3609	1.70 (1.64–1.75)	444	1.29 (1.18–1.42)	< 0.001

*IR incidence rate per 1000 person-years.

^a^No DKD: eGFR ≥ 90 mL/min/1.73m^2^, negative or trace in dipstick proteinuria, ^b^DKD stage 1: eGFR ≥ 90 mL/min/1.73m^2^, ≥ 1 + in dipstick proteinuria, ^c^DKD stage 2: 60 mL/min/1.73m^2^ ≤ eGFR < 90 mL/min/1.73m^2^, ^d^DKD stage 3: 30 mL/min/1.73m^2^ ≤ eGFR < 60 mL/min/1.73m^2^, ^e^DKD stage 4/5: eGFR < 30 mL/min/1.73m^2^.

In the risk assessment, the advanced stage of DKD showed the highest risk of death from all causes, endocrine and metabolic disorders and circulatory system disorders. The risk of all categories of death was significantly increased among subjects with DKD stage 1 compared with those with DKD stage 2 (Table 3).

**Table 3:** Risk of causes of death according to DKD stage.

	Group	Number of patients	Events	HR(95% CI)			
				Model 1	Model 2	Model 3	Model 4
All Cause	No DKD	880,927	69,476	0.69 (0.68–0.69)	1.10 (1.09–1.11)	1.05 (1.04–1.06)	1.05 (1.04–1.06)
	DKD stage 1	51,406	6501	1.12(1.09–1.15)	1.75 (1.70–1.79)	1.66 (1.62–1.70)	1.64 (1.60–1.68)
	DKD stage 2	1,384,982	159,776	1(Ref.)	1(Ref.)	1(Ref.)	1(Ref.)
	DKD Stage 3	257,638	66,850	2.41 (2.39–2.43)	1.44 (1.42–1.45)	1.47 (1.45–1.48)	1.44 (1.43–1.46)
	DKD Stage 4	39,709	8749	1.91 (1.87–1.95)	2.13 (2.08–2.18)	2.14 (2.10–2.19)	2.08 (2.03–2.12)
Neoplasm	No DKD	880,927	25,891	0.74 (0.73–0.75)	1.12 (1.10–1.13)	1.09 (1.07–1.10)	1.08 (1.06–1.09)
	DKD stage 1	51,406	2024	1.01 (0.97–1.06)	1.46 (1.39–1.52)	1.40 (1.34–1.47)	1.41 (1.35–1.48)
	DKD stage 2	1,384,982	54,963	1(Ref.)	1(Ref.)	1(Ref.)	1(Ref.)
	DKD Stage 3	257,638	15,704	1.64 (1.61–1.67)	1.06 (1.04–1.08)	1.07 (1.05–1.09)	1.07 (1.05–1.09)
	DKD Stage 4	39,709	1587	1.01 (0.96–1.07)	1.11 (1.06–1.17)	1.12 (1.06–1.18)	1.10 (1.05–1.16)
Endocrine and metabolic disease	No DKD	880,927	4487	0.59 (0.57–0.61)	0.96 (0.93–0.99)	0.90 (0.87–0.93)	0.91 (0.88–0.94)
	DKD stage 1	51,406	684	1.56 (1.44–1.68)	2.52 (2.33–2.73)	2.39 (2.22–2.59)	2.30 (2.13–2.49)
	DKD stage 2	1,384,982	12,101	1(Ref.)	1(Ref.)	1(Ref.)	1(Ref.)
	DKD Stage 3	257,638	8507	4.05 (3.94–4.17)	2.31 (2.25–2.38)	2.39 (2.32–2.45)	2.28 (2.21–2.34)
	DKD Stage 4	39,709	1855	5.37 (5.11–5.64)	6.01 (5.72–6.31)	6.01 (5.73–6.31)	5.69 (5.41–5.97)
Diseases of the circulatory system	No DKD	880,927	11,121	0.56 (0.54–0.57)	0.95 (0.93–0.97)	0.92 (0.90–0.94)	0.93 (0.91–0.95)
	DKD stage 1	51,406	1204	1.05 (0.99–1.11)	1.79 (1.68–1.89)	1.72 (1.62–1.82)	1.64 (1.55–1.74)
	DKD stage 2	1,384,982	31,597	1(Ref.)	1(Ref.)	1(Ref.)	1(Ref.)
	DKD Stage 3	257,638	16,452	3.00 (2.95–3.06)	1.65 (1.61–1.68)	1.67 (1.63–1.70)	1.58 (1.55–1.62)
	DKD Stage 4	39,709	1909	2.11 (2.01–2.21)	2.37 (2.26–2.48)	2.38 (2.27–2.49)	2.22 (2.12–2.33)
Diseases of the respiratory system	No DKD	880,927	5309	0.56 (0.54–0.58)	1.11 (1.08–1.15)	1.04 (1.00–1.07)	1.03 (1.00–1.06)
	DKD stage 1	51,406	473	0.87 (0.79–0.95)	1.70 (1.55–1.87)	1.62 (1.48–1.77)	1.61 (1.47–1.77)
	DKD stage 2	1,384,982	15,046	1(Ref.)	1(Ref.)	1(Ref.)	1(Ref.)
	DKD Stage 3	257,638	6529	2.52 (2.45–2.60)	1.26 (1.23–1.30)	1.31 (1.28–1.35)	1.30 (1.27–1.34)
	DKD Stage 4	39,709	533	1.22 (1.12–1.33)	1.40 (1.29–1.53)	1.41 (1.29–1.53)	1.37 (1.26–1.49)
Diseases of the digestive system	No DKD	880,927	4664	1.16 (1.11–1.20)	1.51 (1.45–1.57)	1.37 (1.31–1.42)	1.35 (1.30–1.40)
	DKD stage 1	51,406	474	2.05 (1.86–2.25)	2.55 (2.32–2.80)	2.22 (2.02–2.43)	2.23 (2.03–2.45)
	DKD stage 2	1,384,982	6361	1(Ref.)	1(Ref.)	1(Ref.)	1(Ref.)
	DKD Stage 3	257,638	2308	2.08 (1.99–2.18)	1.56 (1.49–1.64)	1.64 (1.56–1.72)	1.65 (1.57–1.73)
	DKD Stage 4	39,709	305	1.69 (1.51–1.89)	1.80 (1.60–2.02)	1.85 (1.65–2.08)	1.82 (1.62–2.05)

*DKD* diabetic kidney disease; *HR* hazard ratio; *95% CI* 95% confidence interval.

Model 1 was non-adjusted. Model 2 was adjusted for age and sex. Model 3 was adjusted for variables in model 2 and BMI, smoking history, alcohol consumption and physical activity Model 4 was adjusted for variables in model 3 and comorbidities (hypertension, dyslipidemia, COPD, cancer, and CHF).

### Impact of albuminuria on each cause of mortality

**Figure 2:**
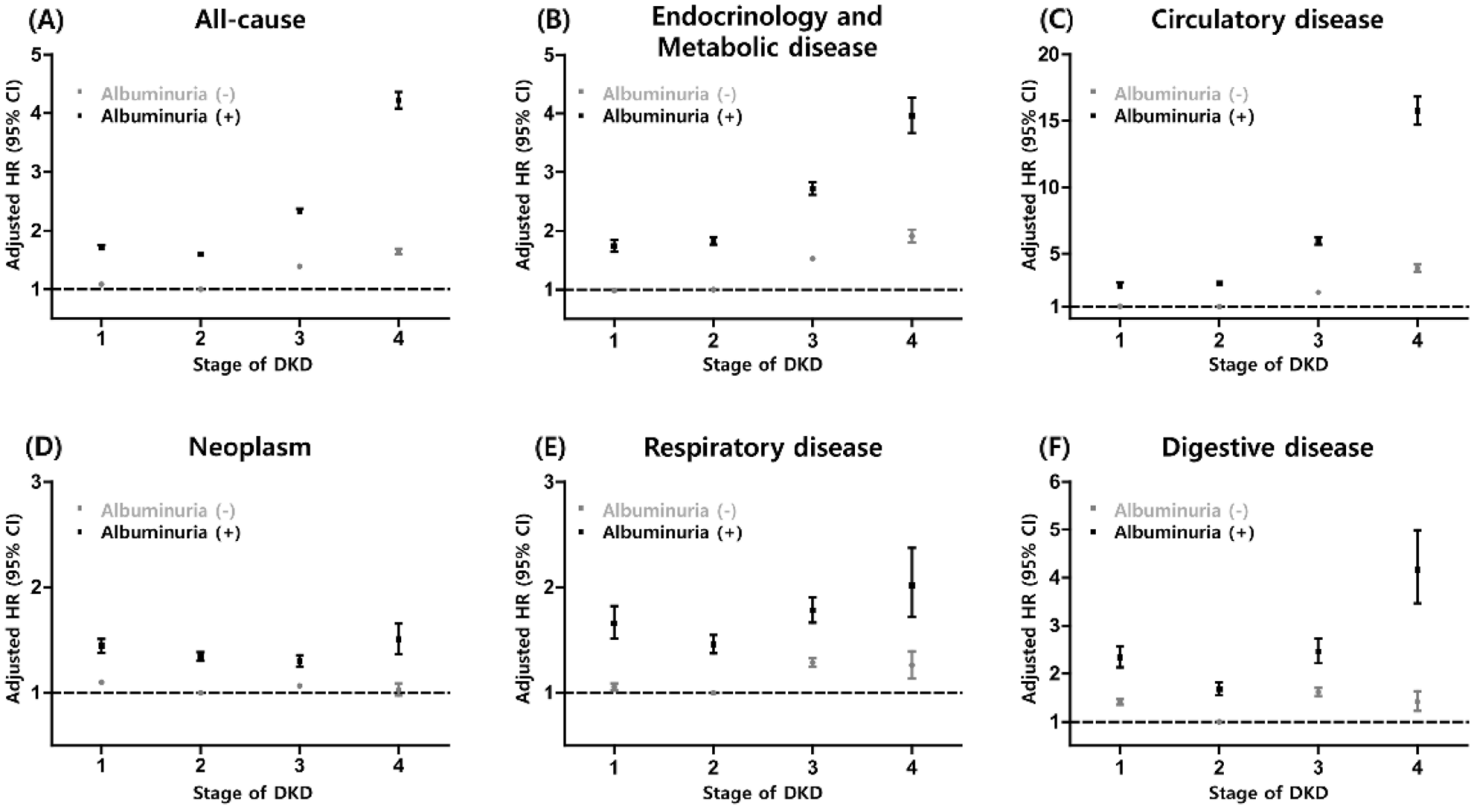
The adjusted risk of mortality from (**A)** all causes, (**B**) endocrinology and metabolic diseases, (**C**) circulatory diseases, (**D**) neoplasms, (**E**) respiratory diseases, and (**F**) digestive diseases according to the stage of diabetic kidney disease. Adjusted variables were age, sex, body mass index, smoking history, alcohol consumption, physical activity and comorbidities such as hypertension, dyslipidemia, chronic obstructive pulmonary disease, cancer, and congestive heart failure. *HR* hazard ratio; *CI* confidence interval; *DKD* diabetic kidney disease.

Even in the same eGFR group, the risk for mortality due to each cause was higher among the subjects with albuminuria (Fig. 2). In the absence of albuminuria, reduced eGFR progressively increased mortality from all causes, endocrine and metabolic diseases, and circulatory diseases. The presence of albuminuria in addition to reduced eGFR exponentially increased the risk of death in these groups (Fig. 2). Death from endocrine and metabolic diseases, the main causes of mortality, was prominently distinguished according to the presence of albuminuria in DKD stage 3 (adjusted HR [aHR], 5.953; 95% CI, 5.687‒6.232 in nonalbuminuria vs. aHR, 2.087; 95% CI, 2.020‒2.156 in albuminuria) and DKD stage 4 (aHR, 15.731; 95% CI, 14.714‒16.819 in nonalbuminuria vs. aHR, 3.887; 95% CI, 3.630‒4.163 in albuminuria).

Although albuminuria in advanced DKD showed a significant impact on the increased risk of mortality from respiratory disease and digestive disease, a reduced eGFR alone did not promote an incrementally increased risk for mortality. Likewise, death from neoplasms was influenced only by the presence of albuminuria, not by reduced eGFR (Fig. 2).

We analyzed the causes of death from the circulatory system in detail. As shown in Supplemental Fig. 1, the risk of death was higher among the subjects with albuminuria than among those without albuminuria in the same stage of DKD. This trend was observed in subcategories of the circulatory system, such as hypertensive disorder, ischemic heart disease, and cerebrovascular disease.

## Discussion

WE investigated the impact of the presence of diabetes on each cause of mortality using national health insurance data in South Korea. Diabetes has a significant role in the death of patients with major organ diseases. Moreover, we identified that the presence of albuminuria significantly increased the risk of mortality irrespective of DKD stage. In particular, the presence of albuminuria adds to the reduced eGFR and synergistically increased mortality from all causes, endocrine and metabolic diseases, and circulatory diseases. Considering the different impacts of eGFR and albuminuria on each cause of mortality, these results could be a helpful guide for the focus of disease management.

Diabetes is a metabolic disorder that is characterized by hyperglycemia and glucose intolerance. Many complications begin with the chronic hyperglycemic status, and this is accompanied by high mortality and morbidity due to microvascular and macrovascular complications^22^. These vascular complications ultimately involve overall major organ diseases, which might increase the risk of mortality irrespective of the site specificity. In this regard, diabetes is associated with not only cardiovascular death but also other substantial premature deaths from cancer, infectious diseases, external causes, and degenerative disorders^23,24^. We found that these risks of mortality were incrementally increased according to kidney dysfunction among patients with diabetes. In addition, the presence of albuminuria has a more prominent impact on the increased risk of mortality irrespective of the cause of death.

Albuminuria is a significant early sign that indicates kidney damage in diabetes. In addition, it has also been recognized as a powerful risk factor for adverse clinical outcomes in various clinical settings, including cardiovascular disease^17,18,25^. Even a small increase in albuminuria or detection of albuminuria at a single visit significantly increased the risk of major cerebro-cardiovascular events such as cardiovascular death, ischemic stroke, and myocardial infarction^26^. In addition to the presence of albuminuria, reduced eGFR is another independent risk factor for cardiovascular and renal outcomes, so these two variables are considered more suitable for risk assessment than other clinical risk factors in diabetic patients^14,27^. Although there are limited data to represent the association between these factors and adverse outcomes other than cardiovascular outcome, this study showed a difference in the impact of albuminuria and reduced eGFR according to each cause of death.

The impact of diabetes on the development of cardiovascular disease and cardiovascular mortality has been extensively evaluated for decades^14,17^. Likewise, similar results were obtained in this study. In particular, the synergistic effect of reduced eGFR and the presence of albuminuria was prominent in death due to circulatory diseases in this study. This finding could be related to the strong association between the severity of DKD and cardiovascular disease. Additionally, we found that subcategories of cardiovascular disease such as hypertensive disorders, ischemic heart disease, other heart disease, and cerebrovascular disease also showed a well-discriminated risk of mortality according to the presence of albuminuria and stage of DKD. Thus, additional attention to albuminuria is necessary for high-risk patients with cardiovascular disease.

In addition to cardiovascular mortality, death from endocrine and metabolic diseases also showed a synergistically increased risk of mortality according to the DKD stage and the presence of albuminuria. Diseases of the endocrine system include not only diabetes and thyroid diseases but also malnutrition, electrolyte imbalances, and acid–base disorder, which could be a common cause of death among patients with advanced kidney disease. However, because the death certificate is completed according to the ICD-10 code, this category could include overall death from diabetes, without evaluation for specific causes. Therefore, a more detailed evaluation of the cause of death in diabetic patients needs to be performed using a well-designed prospective cohort study to improve the accuracy of these vague results.

Interestingly, subjects with DKD stage 1 showed a higher risk of mortality than those with DKD stage 3 for all types of death. Moreover, even subjects with no DKD showed a higher risk of all-cause mortality, death from neoplasms, and death due to diseases of the digestive system than those with DKD stage 2. This could be related to the hazard effect of glomerular hyperfiltration as an independent risk factor for all-cause mortality^28^. Moreover, this relationship was more prominent in diabetic patients, and the risk of mortality in subjects with hyperfiltration was reported to be similar to or even higher than that among subjects with an eGFR < 60 mL/min/1.73 m^2^^29^. However, in the separate analysis according to the presence of albuminuria, the impact of hyperfiltration was attenuated. The risk with DKD stage 1 was significantly higher than that with DKD stage 2 but not higher than that with DKD stage 3 for most causes of death except neoplasms. Based on the results of this study, we suggest that the presence of albuminuria synergistically increased the hazard effect of hyperfiltration for all types of death.

Among the indicators representing kidney dysfunction, in addition to the eGFR value, the presence of albuminuria, a kidney damage marker, showed a more prominent impact on the risk of mortality irrespective of specific causes. Moreover, these two markers showed different associations according to each cause of death. This significant finding, based on the nationwide population cohort, has not been commonly identified before. However, there were several limitations of this study to be discussed. First, this study is a retrospective cohort study. Second, we used only the ICD-10 code on the death certificate to determine the cause of mortality. This means that the exact clinical situation at the time of death could not be identified. Third, only qualitative results for albuminuria were used for analysis, and quantitative results were not available in this study. Fourth, despite the study targeting diabetic patients, we could not consideration of the severity of diabetes, disease duration, number of medications, usage of insulin, family history, or presence of complications. Lastly, this study was conducted based on the large database, it could be related to the smaller *P* value and a higher likelihood of rejecting the null hypothesis. In this regard, we additionally provided 95% CI as a measure to let the readers evaluate the significance of the findings.

The mortality risk among patients with DM was incrementally increased according to the stage of DKD regardless of the cause of death. In addition to kidney function, the impact of albuminuria on mortality was prominent in all stages of DKD. Even for patients with a favorable eGFR, the presence of albuminuria should be considered a significant marker for mortality.

## Materials and methods

### Ethical considerations

This study complied with the Declaration of Helsinki. The institutional review board of Seoul National University Hospital (E-2107-186-1237) approved this study. The attending government organization approved using the data from the National Health Insurance Service (NHIS) (No. NHIS-2021-1-592). The subject data were anonymized and deidentified for the analysis, so the requirement for informed consent was waived.

### Study population and data collection

We extracted subjects with diabetes from the subjects who underwent national health examinations from January 2009 to December 2012. Diabetic patients were identified by the following clinical criteria: (1) fasting plasma glucose ≥ 126 mg/dl at the national health examination and (2) the presence of a claim for the diagnostic codes E11, E12, E13, and E14 based on the 10th International Classification of Diseases (ICD-10) code and the prescription of oral hypoglycemic agents or insulin for diabetes at the same time.

The exclusion criteria were as follows: (1) age < 20 years old, (2) previous treatment with hemodialysis, peritoneal dialysis, or kidney transplantation. The subjects were followed up until December 2019.

### Data collection

All the data were obtained from the National Health Insurance Database of South Korea operated by NHIS. We collected the demographic characteristics, including age, sex, smoking history, alcohol consumption, physical activity, and income level. In addition, we obtained anthropometric data, including body mass index (BMI) and systolic and diastolic blood pressure, which were measured at the time of the routine health examination. Major comorbidities, including hypertension, dyslipidemia, chronic obstructive pulmonary disease, cancer, and congestive heart failure, were also investigated based on the ICD-10 codes. We obtained laboratory results, including serum creatinine, albuminuria, total cholesterol, high-density lipoprotein cholesterol, and low-density lipoprotein cholesterol.

### Definition of DKD stages

The definition and stage of DKD were based on the Kidney Disease: Improving Global Outcomes CKD guideline^30^. The estimated GFR (eGFR) was calculated by the Modification of Diet in Renal Disease equation, and the presence of albuminuria was defined as dipstick urine albumin ≥ 1 + . The stage of DKD was categorized according to the following criteria: (1) no DKD; eGFR ≥ 90 mL/min/1.73 m^2^ and absence of albuminuria, (2) DKD stage 1; eGFR ≥ 90 mL/min/1.73 m^2^ and presence of albuminuria, (3) DKD stage 2; 60 ≤ eGFR < 90 mL/min/1.73 m^2^, (4) DKD stage 3; 30 ≤ eGFR < 60 mL/min/1.73 m^2^, (5) DKD stage 4; eGFR < 30 mL/min/1.73 m^2^. To evaluate the clinical significance of albuminuria in DKD patients, we further categorized the subgroups according to albuminuria in each stage of DKD. The presence of albuminuria was defined as 1 + and above in dipstick urinalysis.

### Study outcomes

The impact of diabetes on each cause of mortality according to the stage of DKD was set as a primary outcome. In addition, we evaluated the impact of albuminuria on each cause of mortality in each stage of DKD. The causes of death were classified into 9 groups according to the ICD-10 code on the death certificate: (1) infectious disease, (2) neoplasm, (3) endocrine and metabolic diseases, (4) nervous system disorders, (5) diseases of the circulatory system, (6) diseases of the respiratory system, (7) diseases of the digestive system, (8) diseases of the genitourinary system, and (9) injury from external causes. We excluded the categories with a very low number of deaths, such as diseases of the eye and adnexa; ear and mastoid process diseases; pregnancy, childbirth and the puerperium; and congenital malformation, deformations, and chromosomal abnormalities, from the analysis.

We additionally evaluated the specific cause of death of subjects with circulatory system disease codes. The detailed causes of death were classified based on the ICD-10 code in detail: (1) hypertensive disorder, (2) ischemic heart disease, (3) other cardiac diseases, (4) cerebrovascular disease, and (5) atherosclerosis.

### Statistical analysis

Continuous variables are presented as the mean ± standard deviation, and categorical variables are presented as percentages. To compare the groups, we performed ANOVA or the chi-square test. Two-sided *P* values were derived by setting the significance level at 0.05. The incidence rate of outcome was demonstrated as per 1000 person-years. For the risk assessment of each cause of death, we selected clinically important and frequent categories for further analysis to determine the significance of albuminuria on mortality: (1) all-cause death, (2) cancer, (3) endocrine and metabolic disease, (4) circulatory system disorders, (5) respiratory system disorders, and (6) digestive system disorders. Cox proportional hazards regression models were used to evaluate the risk of mortality. We adjusted for the following variables: age, sex, BMI, smoking status, drinking status, physical activity, history of comorbidities: hypertension, dyslipidemia, congestive heart failure, chronic obstructive lung disease, and cancer in the multivariate models. The SAS 9.4 program (SAS Institute, Cary, NC, USA) was used to perform statistical analysis.

## Supplementary Information

Supplementary Information.

## Data Availability

The datasets generated and/or analyzed in the current study are available from the corresponding author on reasonable request.
