# Supplementary material for: Impact of albuminuria on the various causes of death in diabetic patients: a nationwide population-based study
Source: Sci Rep. 2023 Jan 6;13:295. doi: 10.1038/s41598-022-23352-0 (PMC9822964; doi:10.1038/s41598-022-23352-0)

**Supplemental Figure 1.** The adjusted risk of each cause of cardiovascular mortality according to the stage of diabetic kidney disease

Adjusted variables were age, sex, body mass index, smoking history, alcohol consumption, physical activity and comorbidities such as hypertension, dyslipidemia, chronic obstructive pulmonary disease, cancer, and congestive heart failure.

HR, hazard ratio; DKD, diabetic kidney disease


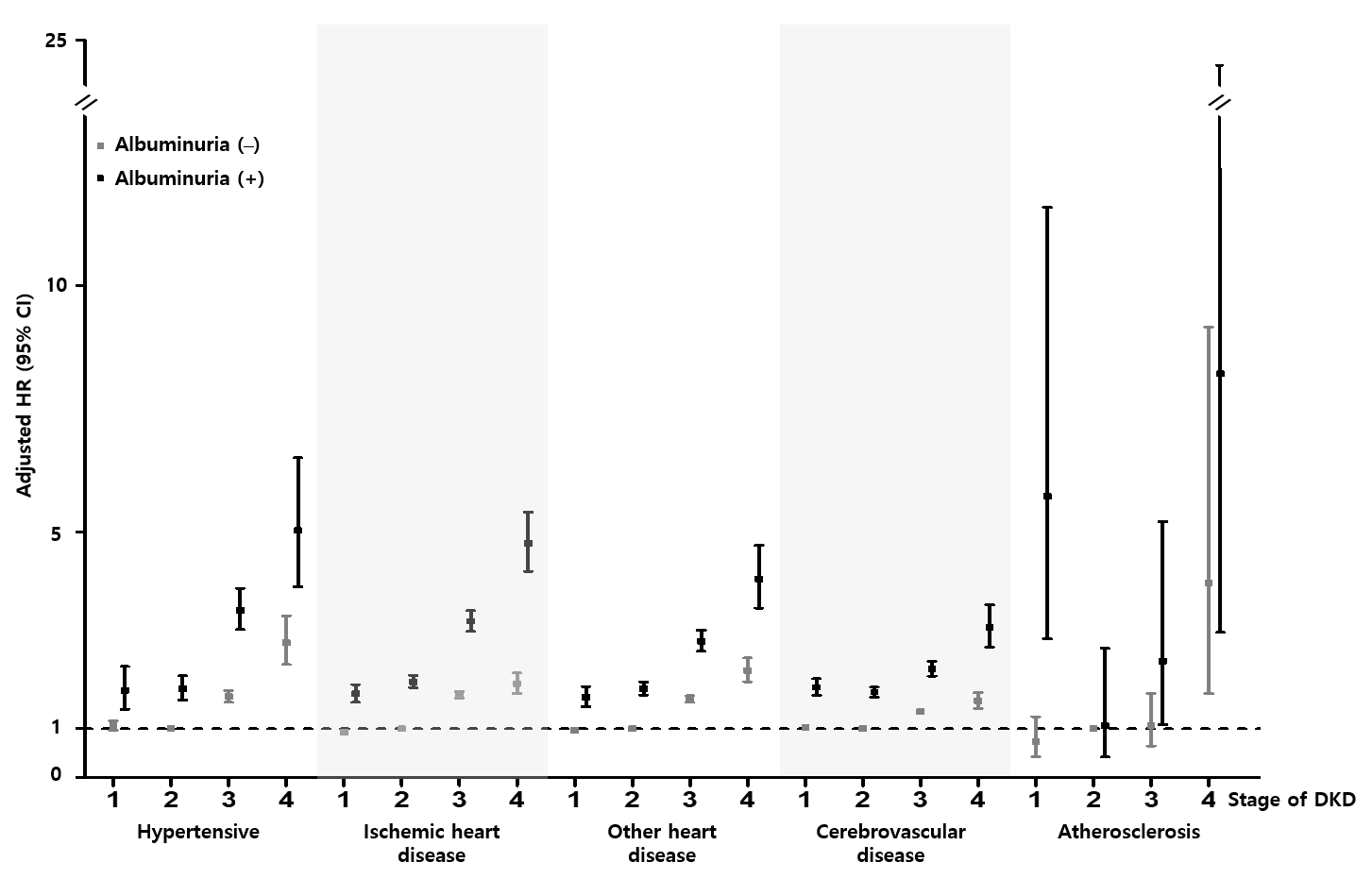

Supplement: Supplementary file 1 — Supplementary Information. [file 41598_2022_23352_MOESM1_ESM.docx]
